# Identification of Antioxidant Plant Extracts and *Garcinia amplexicaulis* Metabolites with Ferroptosis Inhibitor-like Chemical Properties for Alternative Crop Protection

**DOI:** 10.3390/metabo16100714

**Published:** 2026-09-25

**Authors:** Auriane Leblond, Guillaume Viault, Thomas Charpentier, Emilie Roger, Olivier Thomas, Franck Bastide, Thomas Guillemette, Marc Litaudon, Denis Séraphin, Jean-Jacques Hélesbeux

**Affiliations:** 1 SONAS, SFR QUASAV, University of Angers, F-49000 Angers, France; auriane.leblond@univ-angers.fr (A.L.); guillaume.viault@univ-angers.fr (G.V.); denis.seraphin@univ-angers.fr (D.S.); 2 Équipe “Chimie des Substances Naturelles”, BioCIS, CNRS, Faculty of Pharmacy, Université Paris-Saclay, 17 Avenue des Sciences, 91400 Orsay, France; 3 MINT, Inserm, CNRS, SFR ICAT, CHU Angers, University of Angers, Institut Universitaire de France (IUF), F-49000 Angers, France; 4 Institut Agro, University Angers, INRAE, IRHS, SFR 4207 QuaSaV, F-49000 Angers, France; 5 ICSN Institut de Chimie des Substances Naturelles, CNRS-ICSN, UPR 2301, Université Paris-Saclay, F-91198 Gif-sur-Yvette, France

**Keywords:** plant extracts screening, antifungal activity, crop protection, *Alternaria brassicicola*, δ-amplexichromanol

## Abstract

Background: Sustainable strategies to control fungal diseases are urgently needed due to the environmental and human health impacts of conventional fungicides and the rise in fungicide resistance. Ferroptosis modulation, an iron-dependent programmed cell death, could represent an alternative strategy for crop protection, although its potential for fungal disease management remains largely unexplored. Methods: The aim of this study was to run preliminary screening assays, identify plant extracts and natural products with requested chemical properties for ferroptosis inhibition, and evaluate their ability to limit *Alternaria brassicicola* infection. Results: The CAT-based screening of 165 plant extracts led to the identification of several antioxidant-active extracts, including *Garcinia amplexicaulis* (*G. amplexicaulis*) (bark, DCM), *Garcinia kola* (*G. kola*) (nut, DCM), *Magnolia sieboldii* (*M. sieboldii*) (leaf, MeOH) and *G. amplexicaulis* (leaf, MeOH). The major constituents, δ-amplexichromanol, δ-garcinoic acid, sieboldin and (−)-epicatechin, found in the most active fractions from these extracts displayed CAT activities comparable to those of the synthetic ferroptosis inhibitors ferrostatin-1 and phenoxazine. Among these compounds, δ-amplexichromanol showed detectable in vitro inhibitory activity against *A. brassicicola* and significantly reduced lesion development in planta, with an efficacy comparable to ferrostatin-1 and phenoxazine. The antifungal activity of *G. amplexicaulis* (leaf, MeOH) observed in vitro was also confirmed in planta. Conclusions: these findings highlighted δ-amplexichromanol and *G. amplexicaulis* (leaf, MeOH) as candidates for further investigation and supported the potential of CAT-based antioxidant screening to prioritize plant extracts for subsequent evaluation against *A. brassicicola*.

## 1. Introduction

In the context of global population growth and increasing food demand, phytopathogenic fungi pose a significant threat to global food security by causing major agricultural and economic damage [1,2]. Annual crop losses associated with fungal diseases have been estimated at 10–23% in the field, with an additional 10–20% occurring during storage, resulting in economic costs amounting to billions of dollars worldwide [1]. In response, chemical fungicides were widely used and led to harmful impacts on the environment and human health, along with increased fungicide resistance [1]. Therefore, the development of safer and more effective antifungal alternatives for sustainable plant protection has become a priority, as highlighted by the implementation of several international strategies, such as the United Nations Sustainable Development Goals (SDGs) and the European Green Deal, which aims to promote sustainable agriculture [1,2,3].

Traditional antifungal agents primarily function by disrupting cell wall synthesis, inhibiting membrane sterol biosynthesis, or blocking nucleic acid metabolism [4]. Consequently, current innovative antifungal strategies focus on novel cellular targets and biological mechanisms to develop alternative antifungal approaches, such as dihydroorotate dehydrogenase, oxysterol-binding proteins, melanin biosynthesis, or the unfolded protein response (UPR) [1,5].

Among other emerging mechanisms, ferroptosis is a distinct form of regulated, iron-dependent cell death pathway that was first described in mammalian cells by Dixon et al. [6]. Triggered by the inactivation of glutathione-dependent antioxidant defenses and the iron-driven accumulation of lipid hydroperoxides, ferroptosis can be inhibited by iron chelators and lipophilic antioxidants [7,8]. Since 2017, ferroptosis has also been reported in plants under abiotic and biotic stress conditions [8,9].

Distéfano et al. revealed that heat shock (55 °C) in *Arabidopsis thaliana* root hair cells triggered a cell death with the morphological and biochemical characteristics of ferroptosis, which could be prevented by specific ferroptosis inhibitors, including ferrostatins, lipid peroxidation inhibitors and iron chelators [9].

Then, Dangol et al. showed that the hypersensitive response (HR) in rice plants (*Oryza sativa*) in response to an avirulent or incompatible isolate of the fungal pathogen *Magnaporthe oryzae* also involved ferroptosis. Elevated levels of reactive oxygen species (ROS) and iron were detected in infected rice cells, which could be inhibited by the iron chelator deferoxamine and the radical-trapping antioxidant ferrostatin-1 [8].

In addition, recent studies have highlighted the implication of ferroptosis in a limited number of plant-pathogen interactions, where it appeared to play a role in plant immunity and fungal virulence. In particular, *M. oryzae* required ferroptosis for conidial development, a process essential for the formation of appressoria, specialized infection structures necessary for successful host colonization. Treatments with iron chelators (ciclopirox, deferoxamine), and lipophilic antioxidants (liproxstatin-1, tangeretin), were shown to inhibit this infection process [10,11].

Moreover, ferroptosis has also been observed in postharvest disease of blueberries. Upon infection by *Alternaria alternata* and *Fusarium fujikuroi*, the development of necrotic lesions was associated with increased levels of hydrogen peroxide (H_2_O_2_) and ferrous ions (Fe^2+^). Notably, the application of the iron chelator deferoxamine mesylate significantly reduced lesion expansion [12].

These findings suggest that harnessing ferroptosis inhibition could be a promising alternative strategy for protecting crops against fungal infections [4,10]. Nevertheless, its potential remains largely underexplored in other phytopathogenic systems and deserves further investigation.

Among the different types of ferroptosis inhibitors, radical-trapping antioxidants (RTAs) have been well studied in human health and have demonstrated efficacy as ferroptosis inhibitors [7]. The identification of such molecules can be achieved through antioxidant activity assays. The most widely known and commonly used is the 2,2-diphenyl-1-picrylhydrazyl (DPPH) assay. However, although it is easy to perform and convenient, it does not provide relevant information about the reactivity of the antioxidants towards lipid peroxyl radicals (ferroptosis-related lipid species) and it has been demonstrated to poorly predict the potency of ferroptosis inhibitors [13,14]. Thus, other assays such as the FENIX and Conjugated Autoxidizable Triene (CAT) assays have been developed to evaluate the antioxidant activity of molecules in a lipophilic medium, which better mimics the heterogeneous context of biological membranes [13,15].

Both CAT and FENIX are chemical assays based on the inhibition of lipid peroxidation induced by a radical initiator. Although the FENIX assay has shown a good correlation between measured antioxidant activity and EC50 values obtained in cells (R^2^ = 0.84), the CAT assay presents several advantages, as it monitors the degradation of a natural substrate rather than a synthetic fluorophore, relies on commercially available and inexpensive reagents, and has already been applied to plant extracts [13,15]. Therefore, the CAT assay represents a suitable choice for the screening of plant extracts and antioxidant molecules. However, it should be noted that the CAT assay measures lipid autoxidation in a chemical emulsion and is therefore considered a screening tool for preliminary selection of candidate ferroptosis modulators.

Natural products are an important source of structurally diverse bioactive molecules with a wide range of biological activities [16,17]. Although several natural compounds have been reported to modulate ferroptosis in animal systems, their role in plant and fungal systems remains largely unexplored [18,19].

The aim of the current study was to identify plant extracts as a source of antioxidants with ferroptosis inhibitor-like chemical properties and evaluate their relevance for crop protection using the cabbage-*A. brassicicola* pathosystem, in which the involvement of ferroptosis is still unknown. To this end, an antioxidant screening strategy based on the Conjugated Autoxidizable Triene (CAT) assay was used to identify antioxidant plant extracts. Selected extracts were subsequently fractionated to isolate active compounds, and their antifungal activity was assessed, along with reference ferroptosis inhibitors, through in vitro (mycelial growth inhibition) and in planta assays (lesion development on cabbage leaves).

## 2. Materials and Methods

### 2.1. Chemicals

All chemicals and solvents, including dimethyl sulfoxide (DMSO), Brij^®^ 35, Tween^®^ 20, 2,2′-azobis(2-methylpropionamidine) dihydrochloride (AAPH), ferrous sulfate (FeSO_4_), 3-(2-pyridyl)-5,6-diphenyl-1,2,4-triazine-4′,4″-disulfonic acid sodium salt (ferrozine), ethylenediaminetetraacetic acid disodium salt dihydrate (EDTA-Na_2_), tung oil, trolox, absolute ethanol, 2,2-diphenyl-1-picrylhydrazyl (DPPH), chlorogenic acid, and potato dextrose broth (PDB) medium, were purchased from Sigma-Aldrich (Saint-Quentin-Fallavier, France), while phosphate-buffered saline (PBS) was purchased from Thermo Fisher Scientific (Paisley, UK).

### 2.2. Preparation of the Plant Extracts

Dried plant material was ground into a fine powder and subjected to successive cycles of ultrasound-assisted extraction (15 min, 25 °C, solid-to-solvent ratio 1:10 *w*/*v*) using solvents of increasing polarity: cyclohexane (1 cycle), dichloromethane (1 cycle), ethyl acetate (3 cycles) and methanol (3 cycles). After each extraction step, the mixture was filtered through a Celite pad on a sintered funnel. The combined filtrates obtained with the same solvent were pooled and concentrated to dryness under reduced pressure. The resulting crude extracts were stored at −20 °C until further analysis.

The ethanolic green tea extract (*Camellia sinensis*) used as reference was prepared according to Phonsatta et al. [20].

### 2.3. CAT Assay

The antioxidant capacity of extracts and molecules was evaluated using the conjugated autoxidizable triene (CAT) assay developed by Laguerre et al. [15].

Stock solutions of plant extracts and molecules were prepared in DMSO at 2 mg.mL^−1^ and 8 mM respectively. Diluted solutions (8 μg.mL^−1^ for the extracts; 8 μM for any pure molecule) were prepared in phosphate-buffered saline (PBS) and adjusted to 4% DMSO. 50 µL of these solutions were transferred into UV-Star 96-well microplates and pre-incubated at 37 °C for 5 min with orbital shaking (1200 rpm) in a thermostated shaker.

Tung oil was stripped of tocopherol and other polar impurities according to Laguerre et al. [15]. The stability of the resulting oil at 37 °C was monitored by ^1^H NMR over 150 h, confirming the absence of significant degradation. The stripped tung oil (10 mg) was then emulsified in 50 mL PBS containing 34 μM Brij^®^ 35 (Polyoxyl 23 lauryl ether) by stirring for 5 min, followed by homogenization at 13,000 rpm for 90 s using an Ultra-Turrax homogenizer equipped with an 18 G dispersing tool.

A total of 100 μL of the tung oil-in-PBS emulsion was added to each well. The microplate was then immediately prewarmed and shaken, at 37 °C for 1 min at 1200 rpm.

The reaction was initiated by the addition of 50 μL of an ice-cold 4 mM AAPH solution in PBS, and monitored by recording the decrease in absorbance at 274 nm using a FLUOstar^®^ Omega (version 5.70 R2) microplate reader (BMG LABTECH, Offenburg, Germany). Measurements were taken every 3 min for 5 h at 37 °C, with agitation before each reading.

Final concentrations in each 200 µL-well were 0.1 mg.mL^−1^ tung oil, 17 μM Brij^®^ 35, 1 mM AAPH, 2 μg.mL^−1^ extract (or 2 µM solution of the purified molecule), and 1% DMSO. Controls without antioxidants (emulsion alone or emulsion with AAPH), blanks (extract or pure molecule in PBS without reagents), and a 2 μM Trolox standard were included on each microplate. Analyses were performed in triplicate across two independent microplates. Since this emulsion system has not been previously characterized under these conditions, its stability was confirmed in preliminary studies by Static Multiple Light Scattering (SMLS) using a Turbiscan stability analyzer, while droplet size was determined by Dynamic Light Scattering (DLS) with Nanosizer (Supplementary Figures S1 and S2).

Results were expressed as Trolox equivalents (mol Trolox.mol^−1^ molecule or mmol Trolox.g^−1^ extract) based on the area under the curve (AUC) in the presence of the sample compared to the negative control, according to Laguerre et al. [15]. The results were analyzed using one-way analysis of variance (ANOVA), followed by Tukey’s HSD post hoc test, using GraphPad Prism (version 8.0.1). Differences were considered statistically significant at *p* < 0.05.

### 2.4. Iron Chelation Assay

The iron chelation assay was performed according to an adaptation of the protocol described by Santos et al. [21]. This method is based on the formation of a colored complex between ferrous iron (Fe^2+^) and ferrozine, which can be monitored by its absorbance at 562 nm. In the presence of a chelating agent, the formation of this complex is disrupted, resulting in a decrease in absorbance.

Plant extract solutions were prepared at 2.6 mg.mL^−1^ in methanol containing 5.2% DMSO. A volume of 50 μL of this solution was transferred into each well of a 96-well microplate. Then, 160 μL of water and 20 μL of a 0.30 mM FeSO4 solution were added. The microplate was then shaken in a thermostated shaker for 5 min at 800 rpm. The reaction was initiated by the addition of 30 μL of a 0.80 mM ferrozine solution, and the absorbance was recorded at 562 nm using a FLUOstar^®^ Omega microplate reader (BMG LABTECH, Offenburg, Germany) after 5 min. Molecules and plant extracts were tested at final concentrations of 100 µM and 0.5 mg.mL^−1^, respectively. EDTA was used as positive control.

The percentage of Fe^2+^–ferrozine complex formation was calculated as:

$${\mathrm{Fe}}^{2+}\;\mathrm{chelating}\;\mathrm{ability}\;(\%)\;=\;\frac{Abs\;extract-Abs\;blank\;extract}{Abs\;control}\;\times \;\;100$$

where Abs control is the absorbance of the Fe^2+-^ferrozine complex without plant extract, Abs extract the absorbance in the presence of the plant extract, and Abs blank extract the absorbance of the plant extract without Fe^2+^ and ferrozine, used to correct background absorbance. Results were analyzed by ANOVA followed by Tukey’s HSD test (*p* < 0.05).

### 2.5. DPPH Assay

The radical-scavenging activity was evaluated using the 2,2-diphenyl-1-picrylhydrazyl (DPPH) assay as described by Abdel-Lateff et al. and adapted from Boisard et al. [22,23]. Briefly, plant extracts and standards were diluted in absolute ethanol at 0.02 mg.mL^−1^ from 2 mg.mL^−1^ DMSO stock solutions. In a 96-well plate, 100 µL of each sample (in triplicate) was mixed with 75 µL of absolute ethanol. The reaction was initiated by adding 25 µL of a freshly prepared 1 mM DPPH solution using the microplate reader injector (FLUOstar^®^ Omega, BMG LABTECH) to reach a final volume of 200 µL. After 30 min of incubation in the dark at room temperature, absorbance was measured at 517 nm. Ethanol was used as a solvent blank, while an extract blank was prepared to account for the background absorbance of the extracts. Trolox (10–75 µM) was used for the calibration curve, and chlorogenic acid (0.02 mg.mL^−1^) was included as a quality control. Results are expressed as millimoles of Trolox equivalents per gram of dry extract (mmol TE.g^−1^) and analyzed by ANOVA followed by Tukey’s HSD test (*p* < 0.05).

### 2.6. Fractionation of Crude Extracts by Normal Phase Flash Chromatography

Fractionation of the active extract was performed using normal-phase flash chromatography on silica gel (SiO_2_) equipped with UV detection. Detailed chromatographic conditions are provided in Table S2 (Supplementary Information). Fractions were collected based on UV detection at 254 and 296 nm and pooled according to their TLC chromatographic profiles.

### 2.7. In Vitro Fungal Growth Monitoring by Laser Nephelometry

For inoculum preparation, conidia of *A. brassicicola* strain Abra 43 were collected from the 8-day-old solid cultures by adding sterile water, followed by gentle scraping of the agar plates. An aqueous conidial suspension was then prepared and calibrated to 10^6^ conidia.mL^−1^ using a Thoma counting chamber. A volume of 100 μL of this solution was mixed with 890 μL of potato dextrose broth (PDB) medium and 10 μL of the tested compound or extract dissolved in DMSO. The resulting mixture was divided into three 300 μL aliquots and transferred in triplicate into a 96-well microplate (Greiner^®^, polystyrene, flat-bottom, sterile). Final concentrations were 100 μM for the purified molecule, 0.5 mg.mL^−1^ for the extract, and 1% DMSO. Turbidity was measured every 10 min for 33 h using a Nephelostar^®^ Plus (BMG LABTECH, Offenburg, Germany) at 25 °C with orbital shaking. Data were processed as previously described by Joubert et al. [24]. Statistical analyses were performed on the raw AUC data using one-way ANOVA followed by Tukey’s HSD post hoc test in GraphPad Prism. Differences were considered statistically significant at *p* < 0.05.

### 2.8. In Planta Infection Assays

Infection assays were performed on 5-week-old *Brassica oleracea* plants according to Charpentier et al., with slight modifications [5]. Spore suspensions of *A. brassicicola* were prepared at 10^6^ spores.mL^−1^ in water. A total of 50 μL of this solution was mixed with 445 μL of potato dextrose broth (PDB), 5 μL of the tested molecules (10 mM) or plant extracts (50 mg.mL^−1^), and 5 μL of Tween 20 (1%).

Three to four leaves per plant were inoculated with 5 μL droplets of this solution. For each leaf, three controls were also included: a positive control containing the fungus without treatment, a mock control containing the molecules or extracts without fungus, and a negative control containing water with Tween 20 (0.01%) only.

Following inoculation, plants were maintained under saturated humidity in darkness for 48 h, followed by a 3-day incubation under an 8 h light photoperiod in controlled-environment chambers (21 °C and 19 °C for day and night temperatures, respectively).

Disease symptoms were documented by photography, and necrotic lesion areas were quantified using ImageJ (version 1.54p). Three independent biological experiments were carried out.

Statistical analyses were conducted using GraphPad Prism. Necrotic areas obtained with and without inhibitors were compared using the paired Wilcoxon test. Significant differences relative to the untreated condition were indicated as follows: *p* < 0.01 (∗∗), and *p* < 0.001 (∗∗∗).

## 3. Results and Discussion

### 3.1. Antioxidant Screening

A total of 165 extracts obtained from 55 whole plants or plant parts were evaluated for their antioxidant activity using the emulsion-based Conjugated Autoxidizable Triene (CAT) assay (Supplementary Table S1). Plant materials were selected based on material availability and phytochemical interest, including reported antioxidant potential and/or limited phytochemical characterization.

Among them, 57 extracts exhibited higher antioxidant activity than the ethanolic green tea extract used as reference (1.54 mmol Trolox.g^−1^ extract) [20], showing their potential as natural antioxidants. Notably, most of the active extracts belong to plant families known to contain antioxidants. For instance, plants belonging to the Clusiaceae family are characterized by the presence of polyphenols such as xanthones, flavonoids and benzophenones, which have been reported for their antioxidant properties [25,26]. Similarly, extracts from the genus *Malus* are known to contain high levels of polyphenols, including flavonoids, dihydrochalcones and phenolic acids, which could contribute to their observed activity [27]. In addition to polar antioxidants found in EtOAc and MeOH extracts, DCM extracts can contain other lipophilic bioactive constituents, such as terpenoids and prenylated compounds, which may be involved in the observed activity [25,26].

The ten extracts exhibiting the highest antioxidant capacity are presented in Figure 1. These extracts were further evaluated using complementary assays, including DPPH radical scavenging and iron chelation.

*Garcinia amplexicaulis* (leaf, MeOH) and *Malus sieboldii* (leaf, MeOH) extracts showed the highest antioxidant activity in both CAT and DPPH assays, whereas the other extracts displayed lower activity in the DPPH assay compared to the CAT assay (Figure 1A,B). This difference may indicate that the most active compounds in these extracts are more active in lipid environments rather than in homogeneous aqueous systems. This suggests the presence of lipophilic antioxidants, which tend to localize at lipid interfaces and inhibit lipid peroxidation rather than only scavenging free radicals in the aqueous phase. This property is particularly relevant in the context of ferroptosis, since it is driven by lipid peroxidation. Since *G. amplexicaulis* (leaf, MeOH) and *M. sieboldii* (leaf, MeOH) extracts exhibited strong performance in both assays, it indicates that they may contain both lipophilic and hydrophilic compounds, enabling them to inhibit lipid peroxidation while also scavenging hydrophilic radicals.

Ferroptosis is also driven by non-enzymatic chemical mechanisms involving iron-dependent Fenton-type reactions, which promote radical generation and lipid peroxidation [7]. Consequently, the availability of intracellular iron plays a central role in ferroptosis regulation. In this context, iron chelators can limit these oxidative processes by reducing the pool of redox-active iron. Therefore, in addition to radical scavenging activity, the iron-chelating properties of the selected extracts were evaluated (Figure 1C). All extracts exhibited low to moderate iron chelation capacity, with the highest values observed for *G. amplexicaulis* (leaf, MeOH), *Garcinia livingstoneii* (root, DCM), and *Garcinia kola* (nut, DCM) extracts, although lower than the one obtained for the green tea reference extract.

These results suggest that these extracts combine lipid antioxidant activity with moderate iron chelation capacity. Taken together, these properties could enhance their overall protective potential against oxidative damage.

Based on these findings, four extracts were selected for further investigation: the methanolic extracts from *G. amplexicaulis* and *M. sieboldii* leaves, the dichloromethane extract from *G. amplexicaulis* barks, and the dichloromethane extract from *G. kola* nuts. Although root extracts from *Moronobea coccinea* and *G. livingstoneii* were also ranked among the most active samples, they were not selected due to limited raw material availability and less sustainable sourcing compared to the selected nut and bark extracts.

Furthermore, *G. amplexicaulis* (leaf, MeOH) and *G. kola* (nut, DCM) extracts demonstrated both strong antioxidant activity and notable iron chelation properties, suggesting the presence of bioactive compounds acting through both mechanisms.

### 3.2. Fractionation of Selected Active Extracts

The selected extracts were fractionated by flash chromatography. Five fractions were prepared and their antioxidant capacity was evaluated using the CAT assay (Figure 2). Fraction 2 of *G. amplexicaulis* (bark, DCM), *G. kola* (nut, DCM), and *M. sieboldii* (leaf, MeOH) exhibited the highest antioxidant activity, as well as fraction 1 of *G. amplexicaulis* (leaf, MeOH). ^1^H NMR analysis of the active fractions indicated that δ-amplexichromanol (**1**) ref. [28], δ-garcinoic acid (**2**) ref. [28], sieboldin (**3**) ref. [29] and (−)-epicatechin (**4**) ref. [30] were the most abundant constituents in their respective fractions, as confirmed by HPLC analysis (Figures S3 and S4, Supplementary Information). Among these molecules, two were oxidized analogues of vitamin E (**1** and **2**), while the other two (**3** and **4**) were polyphenols containing a catechol group, one of which was glycosylated. Moreover, *G. amplexicaulis* (leaf, MeOH) extract displayed several active fractions, suggesting the presence of additional antioxidant compounds. The antioxidant activity of the purified compounds was also assessed using the CAT assay, revealing strong activity for δ-amplexichromanol, δ-garcinoic acid and (−)-epicatechin comparable to ferrostatin-1 and phenoxazine (known antioxidant ferroptosis inhibitors), and moderate activity for sieboldin (Figure 3). In contrast, the pure molecules showed little to no iron-chelating activity (0–6% inhibition compared with 40% for EDTA at 50 µM), suggesting that the iron-chelating activity observed in crude extracts of *G. amplexicaulis* (leaf, MeOH) and *G. kola* (nut, DCM) is likely due to other constituents.

These results highlight the potential of these extracts as sources of bioactive compounds and justify further investigations to identify additional active molecules, particularly in the *G. amplexicaulis* (leaf, MeOH) extract.

### 3.3. In Vitro Antifungal Assay

The antifungal activity of plant extracts and molecules was determined in vitro using laser nephelometry to monitor the filamentous growth of *A. brassicicola* (Figure 4).

Antifungal activity was observed for the four extracts at 0.5 mg.mL^−1^, with fungal growth inhibition ranging from 33% to 57% (Figure 4A). The green tea extract, used as a reference in the antioxidant screening, also exhibited limited antifungal activity (25% inhibition).

Then, three ferroptosis inhibitors (ferrostatin-1, phenoxazine and deferoxamine) and the four antioxidant compounds identified in the active extracts were tested at 100 µM (Figure 4B).

Ferroptosis inhibitors displayed contrasting results. Phenoxazine, previously described by Shah et al. as a radical-trapping antioxidant with higher activity than ferrostatin-1 (Fer-1) [13], showed strong antifungal activity (93 ± 2% inhibition). In contrast, ferrostatin-1 exhibited only weak activity (15 ± 3%). The iron chelator deferoxamine (DFO), known to inhibit ferroptosis in *M. oryzae* during host infection, showed no significant antifungal effect under our conditions (5 ± 4%). However, it should be noted that the concentration tested in this study was substantially lower than those commonly reported in the literature (typically 1–3 mM), which may account for the lack of observable activity [8,31]. Microscopic examinations revealed a positive correlation between the nephelometry measurement and the conidia germination. In the presence of ferrostatin-1 and deferoxamine, conidia produced branched hyphae and a dense mycelium similar to positive control, whereas spore germination was noticeably reduced with phenoxazine (Figure 4C). In addition, antioxidant compounds isolated from the most active extracts did not exhibit significant antifungal activity, except for δ-amplexichromanol, which showed low inhibition (22 ± 3%).

Overall, these results indicate that antioxidant molecules and ferroptosis inhibitors have no significant effect on *A. brassicicola* fungal growth in vitro, except phenoxazine. This apparent lack of activity raised concerns regarding the relevance of the nephelometric assay for assessing the antifungal effects of potential ferroptosis modulators in *A. brassicicola*. Indeed, ferroptosis-related mechanisms in fungi such as *M. oryzae* are primarily associated with spore germination and the formation of melanized appressoria [11]. However, the in vitro conditions used in this study were optimized for mycelial growth and did not promote the development of appressorium-like structures. Moreover, the absence of environmental stress in the growth medium may not favor the activation of stress response pathways that could potentially involve ferroptosis.

Therefore, to assess whether the lack of activity observed in vitro is related to the experimental conditions rather than the intrinsic properties of the compounds, complementary in planta assays were performed to further investigate the role of antioxidant and anti-ferroptotic molecules in *A. brassicicola* infection.

### 3.4. In Planta Antifungal Assay

The effects of the tested compounds and *G. amplexicaulis* (leaf, MeOH) extract were further evaluated by monitoring the development of necrotic lesions on cabbage leaves caused by *A. brassicicola* (Figure 5). The *G. amplexicaulis* (leaf, MeOH) extract was selected because it displayed the highest antioxidant activity with the CAT assay, along with moderate iron-chelating properties, in vitro antifungal activity and suitable solubility in potato dextrose broth (PDB).

All compounds were tested at the same concentration as the one used in the in vitro antifungal assay (100 μM). Among the molecules evaluated, δ-amplexichromanol (**1**) significantly reduced lesion development caused by *A. brassicicola*, with a 58% decrease in lesion area compared with the untreated control (*p* = 0.002; Figure 5A). Similarly, the RTAs ferroptosis inhibitors ferrostatin-1 and phenoxazine reduced necrotic lesions by 71% (*p* = 0.0001) and 46% (*p* = 0.002) respectively (Figure 5F,G), while the other tested compounds did not significantly affect fungal development in planta.

These results were consistent with the nephelometric assay, as only δ-amplexichromanol, phenoxazine and ferrostatin-1 exhibited in vitro antifungal activity. However, differences in efficacy were observed between the in vitro and in planta assays. Although phenoxazine displayed the strongest antifungal activity in vitro, it was less active in planta than δ-amplexichromanol and ferrostatin-1, which showed only limited antifungal activity in vitro. This difference may suggest distinct modes of action among the compounds, as δ-amplexichromanol and ferrostatin-1 may primarily interfere with *A. brassicicola* host penetration or early infection processes, whereas phenoxazine may mainly affect fungal growth. Moreover, this discrepancy could be related to differences in compound stability and bioavailability within plant tissues, or differences in plant defense responses that may modulate their apparent antifungal efficacy. Further studies would be required to determine the relative contribution of these factors to the observed in planta activity.

Interestingly, *G. amplexicaulis* (leaf, MeOH) extract, which was identified as one of the most active extracts during antioxidant screening and exhibited moderate iron-chelating and antifungal activities in vitro, also significantly reduced lesion development in planta (Figure 5E). In contrast, (−)-epicatechin (**4**), identified as a major antioxidant constituent of this extract, did not significantly affect lesion development when tested individually (Figure 5D). These results could be explained by synergistic interactions with other antioxidant or iron-chelating constituents present in the extract, or by the contribution of additional bioactive compounds from the other fractions that would require further phytochemical exploration. Notably, δ-amplexichromanol was not detected in the leaf extract of *G. amplexicaulis*, as confirmed by HPLC analysis (Supplementary Figure S5).

## 4. Conclusions

This study identified several antioxidant plant extracts through CAT-based screening, complemented by additional antioxidant profile characterization. Among them, the *G. amplexicaulis* (leaf, MeOH) extract exhibited the highest antioxidant activity and showed both in vitro and in planta antifungal activity against *A. brassicicola*. The metabolite δ-amplexichromanol found in the *G. amplexicaulis* (bark, DCM) extract displayed antioxidant activity and in planta antifungal activity against *A. brassicicola*, similar to the synthetic ferroptosis inhibitors ferrostatin-1 and phenoxazine. In contrast, the iron chelator DFO failed to inhibit necrotic lesion development caused by *A. brassicicola* under the tested conditions. Future studies are needed to elucidate the mode of action of the δ-amplexichromanol, as well as those of ferrostatin-1 and phenoxazine in this system, and to determine whether ferroptosis-related mechanisms contribute to their antifungal activity. Finally, these findings provide a basis for further evaluation of these compounds, namely through phytotoxicity assessments and field studies, towards the development of environmentally friendly fungicides for the control of *Alternaria* diseases.

## Figures and Tables

**Figure 1:**
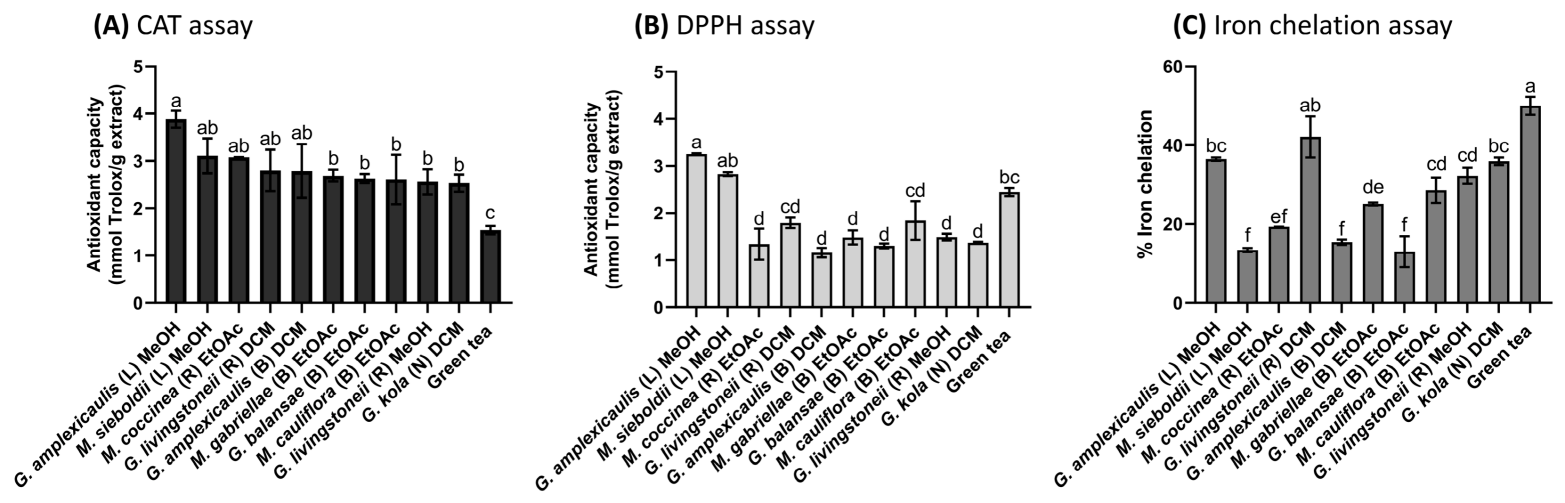
Antioxidant and iron-chelation capacities of the ten most active extracts identified by the CAT screening. (**A**) Antioxidant capacity determined using the Conjugated Autoxidizable Triene (CAT) assay. All extracts were tested at 2 µg.mL^−1^, and results are expressed as Trolox equivalents. (**B**) Antioxidant capacity determined using the 2,2-diphenyl-1-picrylhydrazyl (DPPH) assay. All extracts were tested at 10 µg.mL^−1^, and results are expressed as Trolox equivalents. (**C**) Iron-chelation capacity determined using the iron-chelation assay. All extracts were tested at 0.5 mg.mL^−1^, and results are expressed as percentage of iron chelation relative to the control without chelating agent. L, R, B and N are abbreviations for leaf, root, bark and nut, respectively. Green tea refers to the ethanolic green tea extract used as reference. Letters above bars correspond to homogeneity groups determined using one-way ANOVA followed by Tukey’s HSD multiple comparison test. Conditions sharing the same letter are not significantly different (*p* > 0.05).

**Figure 2:**
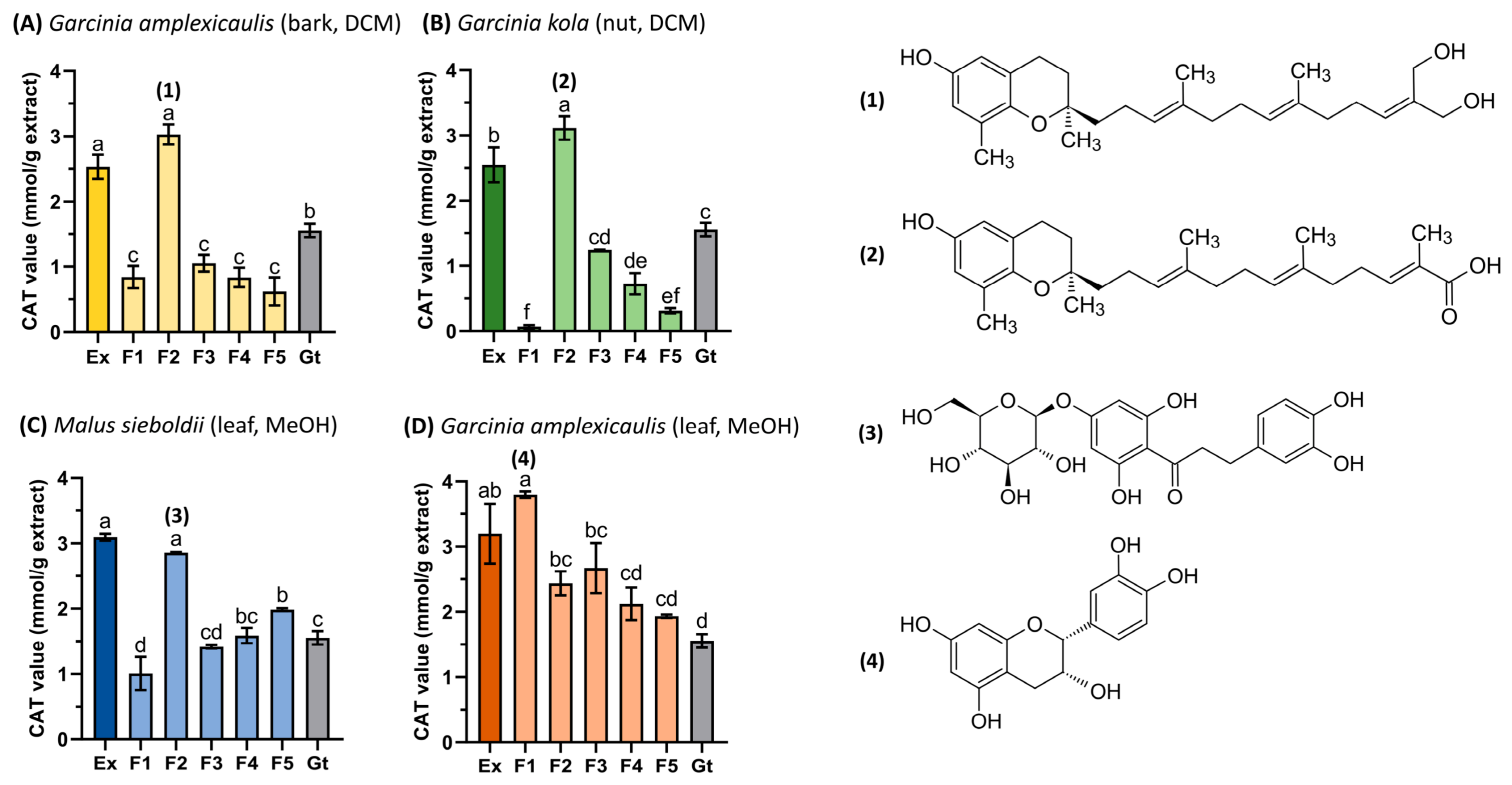
Antioxidant activity of fractionated extracts and structures of major compounds found in active fractions. All fractions were tested at 2 µg.mL^−1^, and results are expressed as Trolox equivalents. Compounds (**1**)–(**4**) correspond to the major constituents present in the respective active fractions: (**1**) δ-amplexichromanol, (**2**) δ-garcinoic acid, (**3**) sieboldin, (**4**) (−)-epicatechin. Ex, F, and Gt refer to total extract, fraction, and green tea reference extract, respectively. NMR analysis was used to confirm the presence of these molecules. Letters above bars correspond to homogeneity groups determined using one-way ANOVA followed by Tukey’s HSD multiple comparison test. Conditions sharing the same letter are not significantly different (*p* > 0.05).

**Figure 3:**
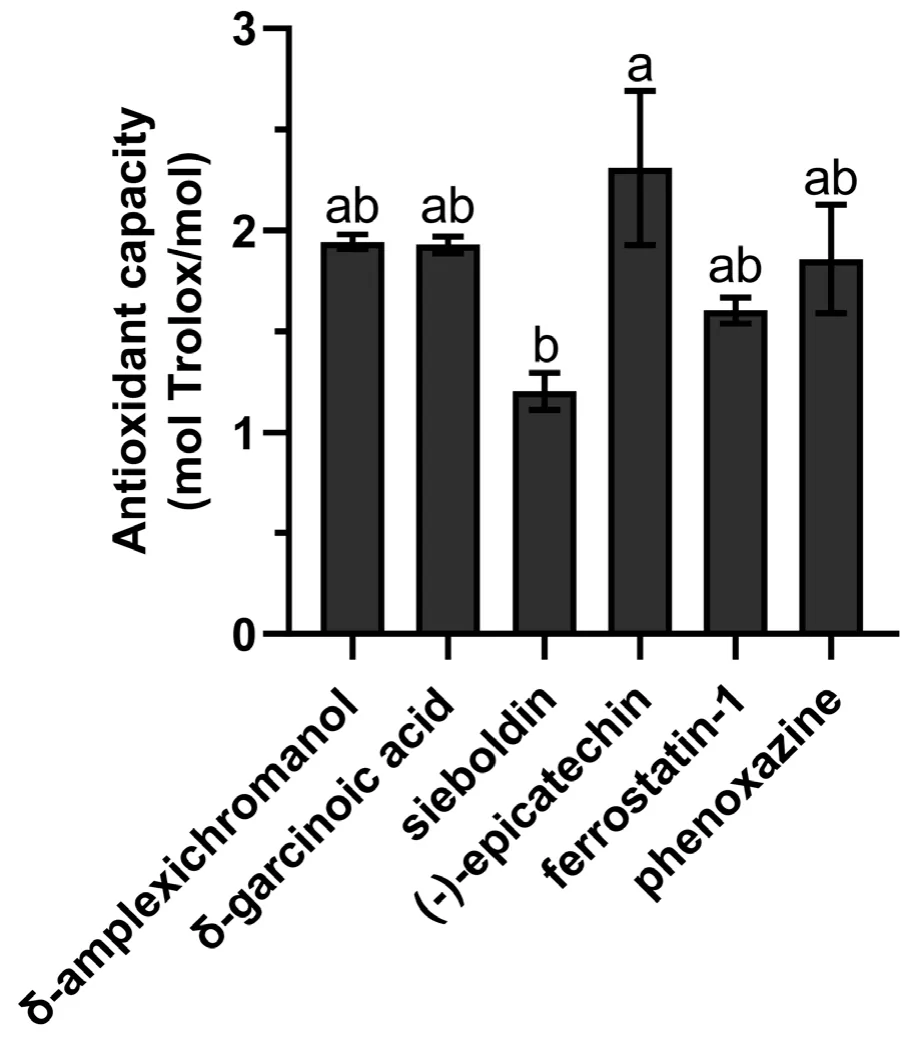
Antioxidant activity assessed with the CAT assay of purified natural products. All molecules were tested at 2 µM, and results are expressed as Trolox equivalents. Ferrostatin-1 and phenoxazine, known ferroptosis inhibitors, were used as reference compounds. Letters above bars correspond to homogeneity groups determined using one-way ANOVA followed by Tukey’s HSD multiple comparison test. Conditions sharing the same letter are not significantly different (*p* > 0.05).

**Figure 4:**
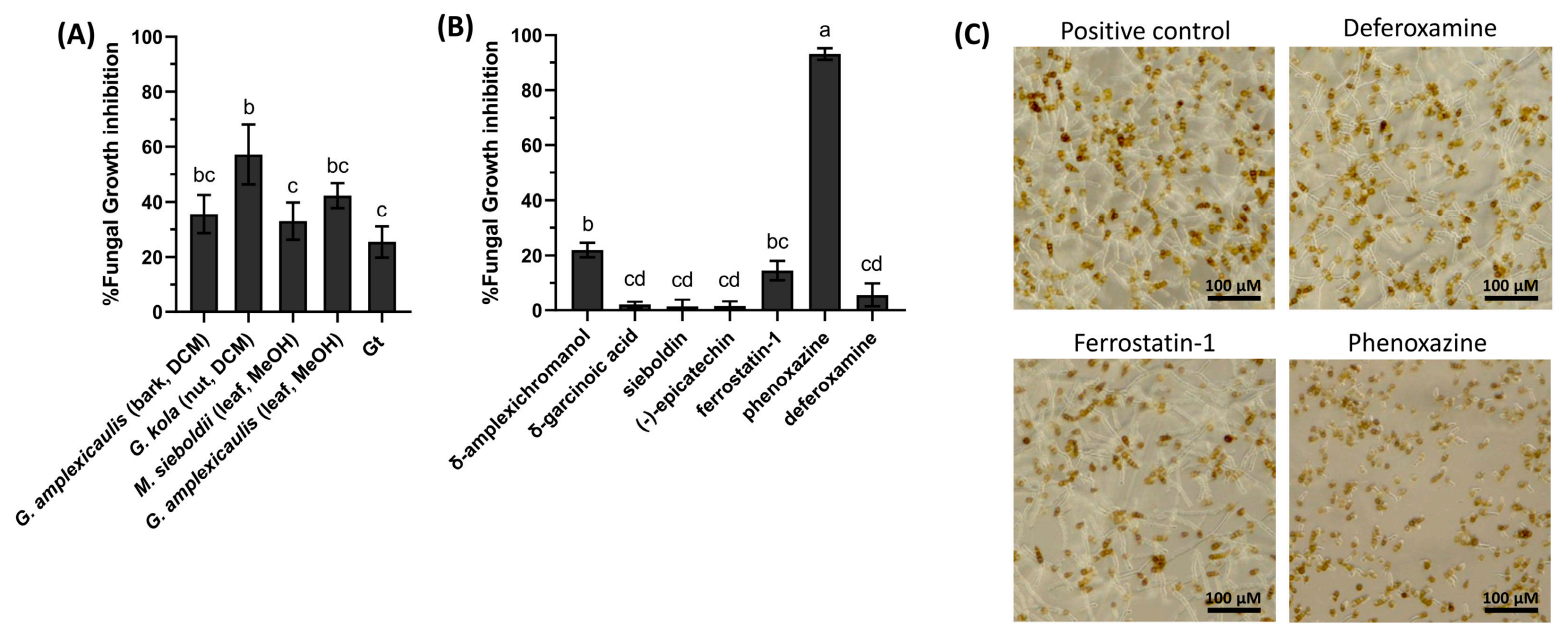
Antifungal activity of extracts, purified compounds, and reference ferroptosis inhibitors on *A. brassicicola* growth. Fungal growth was monitored by laser nephelometry. The percentage of fungal growth inhibition was calculated relative to the positive control (fungus alone). All compounds were tested at 100 µM and extracts at 0.5 mg.mL^−1^. Statistical analyses were performed on the raw AUC data using one-way ANOVA followed by Tukey’s HSD test (Supplementary Table S3). Conditions sharing the same letter are not significantly different (*p* > 0.05). (**A**) Antifungal activity of extracts. (**B**) Antifungal activity of purified natural compounds and reference ferroptosis inhibitors. (**C**) Microscopic observations of *A. brassicicola* conidia following treatment of deferoxamine, ferrostatin-1 and phenoxazine, compared to the positive control without treatment.

**Figure 5:**
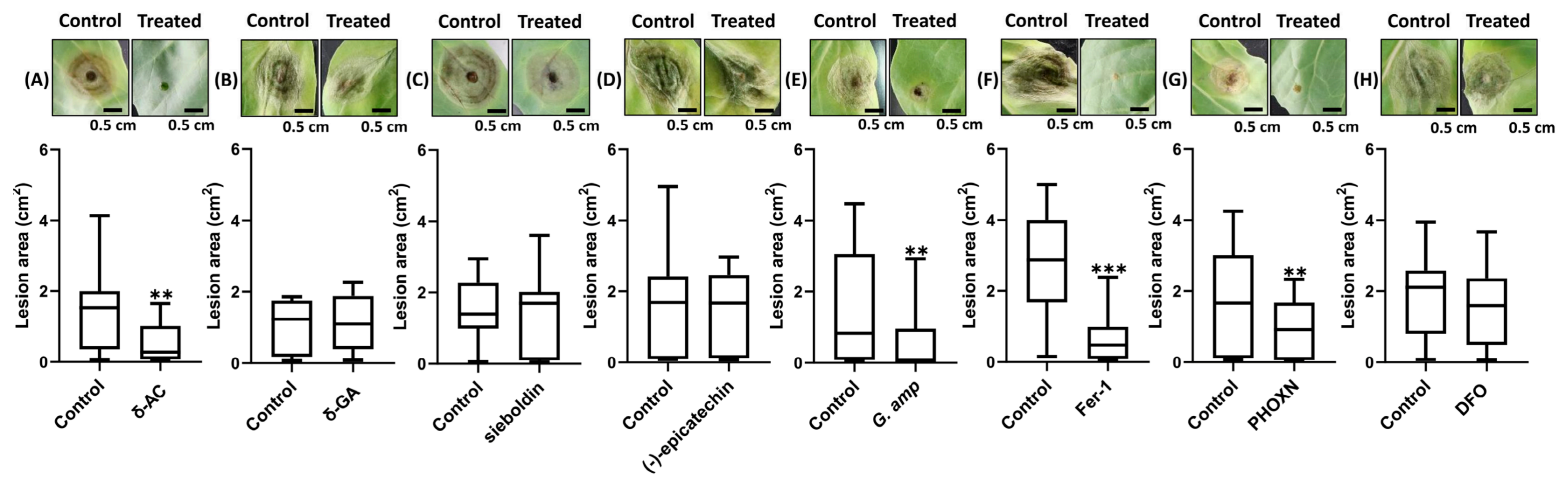
Development of necrotic lesions on cabbage leaves caused by *A. brassicicola*. All compounds were tested at 100 µM and the extract at 0.5 mg.mL^−1^. The “Control” label refers to the untreated fungal inoculum (positive control) and the “Treated” label to the treated fungal inoculum on the same leaf as the positive control. The letters correspond to: (**A**) δ-AC = δ-amplexichromanol, (**B**) δ-GA = δ-garcinoic acid, (**C**) sieboldin, (**D**) (−)-epicatechin, (**E**) *G. amp* = *G. amplexicaulis* (leaf, MeOH) extract, (**F**) Fer-1 = ferrostatin-1, (**G**) PHOXN = phenoxazine, (**H**) DFO = deferoxamine. Data were obtained from three independent biological experiments with *n* = 12 ± 2 replicates. ∗∗ and ∗∗∗ indicate significant differences compared with the positive control, with *p* < 0.01, and *p* < 0.001, respectively, obtained with the paired Wilcoxon test.

## Data Availability

All data generated and analyzed during this study are included in the article and its Supplementary Materials.

## Supplementary Materials

The following supporting information can be downloaded at: https://www.mdpi.com/article/10.3390/metabo16100714/s1, Table S1: Antioxidant capacity of the screened extracts using the CAT assay; Table S2: Methodology for fractionation of active extracts; Table S3: Area under the curve (AUC) values and Tukey grouping for fungal growth monitored by nephelometry; Table S4: Lesion area development caused by *Alternaria brassicicola* on cabbage leaves; Figure S1: Turbiscan Stability Index (TSI) of the tung oil emulsion during the first 8 h; Figure S2: Characterization of the emulsion by dynamic light scattering; Figure S3: ^1^H NMR spectra of the studied fractions; Figure S4: HPLC-UV chromatographic profiles of the most active fractions obtained from the four selected extracts; Figure S5: Comparative HPLC-UV chromatographic profiles of *G. amplexicaulis* (leaf, MeOH) extract, (−)-epicatechin, and δ-amplexichromanol.

## Abbreviations

The following abbreviations are used in this manuscript:

AAPH	2,2-Azobis(2-methylpropionamidine) dihydrochloride
AUC	Area under the curve
CAT	Conjugated autoxidizable triene
DCM	Dichloromethane
DFO	Deferoxamine
DMSO	Dimethyl sulfoxide
DPPH	2,2-Diphenyl-1-picrylhydrazyl
EC50	Half-maximal effective concentration
EDTA	Ethylenediaminetetraacetic acid
EtOAc	Ethyl acetate
Fer-1	Ferrostatin-1
Gt	Green tea
MeOH	Methanol
NMR	Nuclear magnetic resonance
PBS	Phosphate-buffered saline
PDB	Potato dextrose broth
PHOXN	Phenoxazine
TLC	Thin-layer chromatography
RTA	Radical trapping antioxidant
