# Supplementary material for: Identification of Antioxidant Plant Extracts and Garcinia amplexicaulis Metabolites with Ferroptosis Inhibitor-like Chemical Properties for Alternative Crop Protection
Source: Metabolites. 2026 Sep 25;16(10):714. doi: 10.3390/metabo16100714 (PMC13707028; doi:10.3390/metabo16100714)

Supporting Information

Identification of Antioxidant Plant Extracts and *Garcinia amplexicaulis* Metabolites with Ferroptosis Inhibitor-like Chemical Properties for Alternative Crop Protection

Auriane Leblond[1]*, Guillaume Viault[1], Thomas Charpentier[2], Emilie Roger[3], Olivier Thomas[3], Franck Bastide[4], Thomas Guillemette[4], Marc Litaudon[5], Denis Séraphin[1]*, Jean-Jacques Helesbeux[1]*

Table of Contents

Table S1. Antioxidant capacity of the screened extracts using the CAT assay 2

Table S2. Methodology for fractionation of active extracts 6

Table S3. Area under the curve (AUC) values and Tukey grouping for fungal growth monitored by nephelometry 7

Table S4. Lesion area development caused by *Alternaria brassicicola* on cabbage leaves 8

Figure S1. Turbiscan Stability Index (TSI) of the tung oil emulsion during the first 8 h 9

Figure S2. Characterization of the emulsion by dynamic light scattering. 9

Figure S3. ^{1}H NMR spectra of the studied fractions 10

Figure S4. HPLC-UV chromatographic profiles of the most active fractions obtained from the four selected extracts 12

Figure S5. Comparative HPLC-UV chromatographic profiles of *G. amplexicaulis* (leaf, MeOH) extract, (-)-epicatechin, and δ-amplexichromanol 14

Table S1. Antioxidant capacity of the screened extracts using the CAT assay. Results are expressed as Trolox equivalents (mmol.g^{-1} extract).

Plant	Extract	CAT value (TE)	Standard deviation
Montrouziera cauliflora (trunk bark)	DCM	1.14	0.06
	EtOAc	2.61	0.52
	MeOH	2.23	0.06
Garcinia amplexicaulis (trunk and stem bark)	DCM	2.79	0.56
	EtOAc	1.80	0.06
	MeOH	1.81	0.11
Garcinia xantochymus (bark)	DCM	0.36	0.05
	EtOAc	2.07	0.07
	MeOH	1.41	0.10
Calophyllum caledonicum (trunk bark)	DCM	0.50	0.16
	EtOAc	2.01	0.12
	MeOH	2.38	0.17
Garcinia kola (fruit)	DCM	2.53	0.18
	EtOAc	1.54	0.16
	MeOH	0.86	0.15
Garcinia mangostana (pericarp)	DCM	1.18	0.28
	EtOAc	1.30	0.06
	MeOH	0.99	0.24
Hypericum perforatum (leaf)	DCM	0.36	0.22
	EtOAc	0.48	0.23
	MeOH	0.67	0.09
Garcinia scortechinii (bark)	DCM	0.22	0.04
	EtOAc	1.26	0.24
	MeOH	1.16	0.03
Calophyllum wallichianum (stem bark)	DCM	0.18	0.08
	EtOAc	0.09	0.12
	MeOH	1.55	0.28
Calophyllum inophyllum (bark)	DCM	0.24	0.06
	EtOAc	0.49	0.13
	MeOH	2.42	0.79
Calophyllum molle king (leaf)	DCM	0.28	0.06
	EtOAc	2.31	0.41
	MeOH	2.40	0.46
Garcinia virgata (leaf)	DCM	1.58	0.55
	EtOAc	0.78	0.09
	MeOH	0.74	0.01
Montrouziera cauliflora (leaf)	DCM	1.31	0.08
	EtOAc	1.88	0.34
	MeOH	1.54	0.21

Plant	Extract	CAT value (TE)	Standard deviation
Montrouziera gabriellae (leaf)	DCM	1.21	0.12
	EtOAc	2.23	0.53
	MeOH	1.73	0.50
Montrouziera gabriellae (fruit)	DCM	1.70	0.24
	EtOAc	1.83	0.28
	MeOH	1.92	0.40
Monorobea coccinea (seed)	DCM	0.05	0.03
	EtOAc	1.74	0.06
	MeOH	1.42	0.16
Garcinia parvifolia (ripe fruit)	DCM	0.66	0.21
	EtOAc	0.06	0.02
	MeOH	0.05	0.00
Garcinia parvifolia (green fruit)	DCM	0.79	0.18
	EtOAc	0.11	0.08
	MeOH	0.12	0.07
Garcinia balansae (bark)	DCM	0.51	0.37
	EtOAc	2.63	0.10
	MeOH	1.96	0.24
Garcinia xantochymus (leaf)	DCM	0.26	0.01
	EtOAc	1.95	0.09
	MeOH	2.41	0.11
Garciniaxantochymus (fruit)	DCM	1.92	0.36
	EtOAc	1.28	0.04
	MeOH	0.17	0.02
Garcinia amplexicaulis (leaf)	DCM	0.52	0.31
	EtOAc	1.83	0.09
	MeOH	3.89	0.18
Montrouziera gabriellae (trunk bark)	DCM	1.26	0.02
	EtOAc	2.69	0.12
	MeOH	1.86	0.07
Moronobea coccinea (pericarp)	DCM	1.98	0.14
	AcOEt	1.23	0.09
	MeOH	0.24	0.01
Moronobea coccinea (leaf)	DCM	1.20	0.06
	EtOAc	2.08	0.04
	MeOH	1.14	0.00
Moronobea coccinea (stem bark)	DCM	1.97	0.11
	EtOAc	2.50	0.53
	MeOH	2.33	0.00

Plant	Extract	CAT value (TE)	Standard deviation
Moronobea coccinea (root bark)	DCM	1.74	0.05
	EtOAc	3.07	0.00
	MeOH	2.24	0.14
Garcinia livingstoneii (stem)	DCM	0.18	0.05
	EtOAc	2.21	0.14
	MeOH	1.32	0.11
Garcinia livingstoneii (root)	DCM	2.80	0.44
	EtOAc	2.08	0.33
	MeOH	2.56	0.27
Garcinia mangostana (seed)	DCM	0.08	0.02
	EtOAc	0.03	0.00
	MeOH	0.15	0.05
Garcinia nigrolineata (leaf)	DCM	1.24	0.03
	EtOAc	1.18	0.54
	MeOH	0.62	0.50
Garcinia nigrolineata (bark)	DCM	1.53	0.15
	EtOAc	1.44	0.50
	MeOH	2.09	0.16
Garcinia nigrolineata (fruit)	DCM	1.71	0.17
	EtOAc	1.22	0.02
	MeOH	0.21	0.08
Rheedia acuminata (leaf)	DCM	0.87	0.23
	EtOAc	1.29	0.14
	MeOH	1.18	0.08
Rheedia benthamiana (stem bark)	DCM	1.41	0.08
	EtOAc	0.98	0.24
	MeOH	1.96	0.24
Clusia grandiflora (stem)	DCM	0.69	0.04
	EtOAc	0.83	0.03
	MeOH	0.53	0.10
Calophyllum dispar (leaf)	DCM	0.15	0.02
	EtOAc	0.26	0.05
	MeOH	2.12	0.12
Cratoxylum arborecence (leaf)	DCM	0.31	0.02
	EtOAc	1.47	0.20
	MeOH	1.91	0.45
Cratoxylum arborecence (bark)	DCM	0.53	0.33
	EtOAc	0.37	0.06
	MeOH	1.34	0.38
Cratoxylum formosum (leaf)	DCM	0.08	0.01
	EtOAc	1.75	0.22
	MeOH	1.71	0.28

Plant	Extract	CAT value (TE)	Standard deviation
Cratoxylum formosum (bark)	DCM	0.12	0.02
	EtOAc	0.86	0.05
	MeOH	2.07	0.39
Garcinia scortechinii (leaf)	DCM	0.10	0.04
	EtOAc	1.14	0.23
	MeOH	1.57	0.14
Artocarpus rigidus (leaf)	DCM	0.18	0.12
	EtOAc	0.33	0.07
	MeOH	1.00	0.34
Artocarpus rigidus (bark)	DCM	0.07	0.01
	EtOAc	0.50	0.11
	MeOH	0.33	0.12
Mahuera palustris (stem bark)	DCM	0.19	0.03
	EtOAc	0.60	0.15
	MeOH	2.41	0.36
Platonia insignis (leaf)	DCM	1.25	0.08
	EtOAc	1.86	0.45
	MeOH	0.86	0.16
Symphonia glogulifera (leaf and stem)	DCM	0.68	0.06
	EtOAc	1.54	0.09
	MeOH	1.42	0.08
Symphonia sp1 (root bark)	DCM	0.64	0.13
	EtOAc	1.42	0.15
	MeOH	1.65	0.09
Clusia rosea (underground root)	DCM	0.74	0.21
	EtOAc	0.74	0.16
	MeOH	1.38	0.28
Clusia grandiflora (leaf)	DCM	0.67	0.00
	EtOAc	0.37	0.02
	MeOH	0.44	0.08
Garcinia xanthochymus (stem)	DCM	0.19	0.00
	EtOAc	0.91	0.01
	MeOH	0.92	0.01
Clusia rosea (leaf)	DCM	0.38	0.01
	EtOAc	0.50	0.04
	MeOH	1.37	0.07
Clusia rosea (stem)	DCM	0.31	0.06
	EtOAc	0.55	0.14
	MeOH	0.13	0.01
Clusia rosea (aerial root)	DCM	0.88	0.56
	EtOAc	0.48	0.03
	MeOH	0.14	0.08
Malus sieboldii (leaf)	DCM	0.07	0.03
	EtOAc	1.36	0.13
	MeOH	3.11	0.36

Table S2. Methodology for fractionation of active extracts

Extract	Solvent A	Solvent B	Gradient	Recovered fractions
Garcinia amplexicaulis (bark, DCM)	DCM	Acetone	0 to 100% B with 5 holds at 15%, 25%, 50%, 75% and 100% B	F1: 549 mg<br>F2: 118 mg<br>F3: 61 mg<br>F4: 26 mg<br>F5: 14 mg
Garcinia kola (nut, DCM)	Petroleum ether	Acetone	0 to 100% B with 5 holds at 15%, 30%, 45%, 70% and 100% B	F1: 184 mg<br>F2: 311 mg<br>F3: 78 mg<br>F4: 51mg<br>F5: 18 mg
Malus sieboldii (leaf, MeOH)	Chloroform/ EtOAc /MeOH (6/2/1)	EtOAc/MeOH (5/5)	0 to 100% B with 5 holds at 5%, 10%, 40%, 70% and 100% B	F1: 72 mg<br>F2: 512 mg<br>F3: 88 mg<br>F4: 137 mg<br>F5: 42 mg
Garcinia amplexicaulis (leaf, MeOH)	Ethyl acetate	MeOH	0 to 100% B with 5 holds at 0%, 15%, 30%, 45% and 60% B	F1: 118 mg<br>F2: 436 mg<br>F3: 193 mg<br>F4: 90 mg<br>F5: 25 mg

Table S3. Area under the curve (AUC) values and Tukey grouping for fungal growth monitored by nephelometry. C(+) corresponds to the positive control containing the fungus without treatment, whereas C(-) corresponds to the negative control containing potato dextrose broth (PDB) only. AUC 1, AUC 2 and AUC 3 correspond to the mean AUC values obtained from three technical replicate wells in independent experiments 1, 2 and 3, respectively. The following abbreviations are used for the tested molecules: δ-AC = δ-amplexichromanol, δ-GA = δ-garcinoic acid, (-)-epi = (-)-epicatechin, Fer-1 = ferrostatin-1, PHOXN = phenoxazine and DFO = deferoxamine. Means sharing the same letter are not significantly different according to Tukey's HSD test following one-way ANOVA (alpha = 0.05).

A. Controls and treatment with selected plant extracts

**Sample**	**C(+)**	**C(-)**	***G. amplexi-caulis* (bark, DCM)**	***G. kola* (nut, DCM)**	***M. sieboldii* (leaf, MeOH)**	***G. amplexi-caulis* (leaf, MeOH)**	***Green tea, reference extract***
AUC 1	130.0	0.1	75.4	70.0	93.2	76.9	93.0
AUC 2	140.9	0.0	86.8	59.9	98.6	86.5	100.1
AUC 3	159.0	5.1	114.1	50.7	94.1	83.9	128.9
Mean	143.3	1.8	92.1	60.2	95.3	82.4	107.4
SD	14.6	2.9	19.9	9.6	2.9	5.0	19.0
Tukey group	d	a	bc	b	c	bc	c

B. Controls and treatment with individual compounds

**Sample**	**C(+)**	**C(-)**	**δ-AC**	**δ-GA**	**sieboldin**	**(-)-epi**	**Fer-1**	**PHOXN**	**DFO**
AUC 1	124.6	3.5	95.0	120.5	124.3	120.3	101.7	8.7	122.7
AUC 2	125.4	5.7	100.3	123.1	125.2	125.4	110.7	6.2	112.9
AUC 3	136.2	2.4	108.0	134.5	130.4	134.5	117.9	12.4	128.8
Mean	128.7	3.8	101.1	126.0	126.6	126.7	110.1	9.1	121.5
SD	6.4	1.7	6.5	7.5	3.3	7.2	8.1	3.1	8.0
Tukey group	d	a	b	cd	cd	cd	bc	a	cd

Table S4. Lesion area development caused by *Alternaria brassicicola* on cabbage leaves. The positive control was applied on the same leaf as the treated fungal inoculum and corresponds to the lesion development of the fungus without treatment. All compounds were tested at 100 µM and the extract at 0.5 mg.mL^{-1}. Abbreviations are assigned as follows: (1) δ-amplexichromanol, (2) δ-garcinoic acid, (3) sieboldin, (4) (-)-epicatechin, (5) *G. amplexicaulis* (leaf, MeOH) extract, (PHOXN) phenoxazine, (Fer-1) ferrostatin-1, (DFO) deferoxamine. The number of replicates ranged from 10 to 14 depending on the number of leaves on which the control successfully developed. NA indicates missing data for corresponding replicates.

Condition	Lesion area 1 (cm^2)	Lesion area 2 (cm^2)	Lesion area 3 (cm^2)	Lesion area 4 (cm^2)	Lesion area 5 (cm^2)	Lesion area 6 (cm^2)	Lesion area 7 (cm^2)	Lesion area 8 (cm^2)	Lesion area 9 (cm^2)	Lesion area 10 (cm^2)	Lesion area 11 (cm^2)	Lesion area 12 (cm^2)	Lesion area 13 (cm^2)	Lesion area 14 (cm^2)
Positive control	1.896	0.945	1.974	2.061	0.070	0.137	0.437	1.281	4.136	1.794	NA	NA	NA	NA
Treated by (1)	1.011	0.448	1.030	1.653	0.039	0.082	0.122	0.428	0.104	0.050	NA	NA	NA	NA
Positive control	1.676	1.812	1.547	1.855	0.152	1.560	1.785	0.930	0.457	0.282	0.089	0.074	NA	NA
Treated by (2)	2.258	1.833	1.510	1.709	0.129	2.201	1.902	0.691	0.458	0.610	0.379	0.080	NA	NA
Positive control	2.266	1.786	1.112	1.400	0.056	1.534	0.097	0.989	1.323	2.943	2.337	NA	NA	NA
Treated by (3)	2.016	2.036	1.702	1.848	0.046	0.096	0.182	1.948	3.607	0.079	1.658	NA	NA	NA
Positive control	1.909	1.694	1.796	1.621	0.089	0.083	2.513	1.527	2.424	4.950	0.090	NA	NA	NA
Treated by (4)	2.065	1.546	1.677	2.461	0.079	0.077	2.418	1.542	2.964	2.883	0.112	NA	NA	NA
Positive control	1.964	1.413	3.423	1.159	0.079	0.083	0.042	0.061	0.504	4.020	4.472	0.159	NA	NA
Treated by (5)	0.050	0.296	0.037	1.186	0.009	0.057	0.038	0.023	0.010	2.921	2.597	0.046	NA	NA
Positive control	1.942	1.391	1.968	0.056	0.109	0.094	2.886	0.890	3.400	4.253	NA	NA	NA	NA
Treated by (PHOXN)	1.518	1.045	1.493	0.028	0.071	0.038	0.791	0.063	2.185	2.326	NA	NA	NA	NA
Positive control	3.103	2.654	3.236	2.094	4.219	0.178	2.423	2.123	3.651	3.933	4.682	4.991	0.449	0.153
Treated by (Fer-1)	0.856	0.714	0.131	0.983	1.746	0.090	0.049	0.090	0.768	1.021	2.387	0.038	0.247	0.061
Positive control	2.241	0.935	2.302	1.507	2.536	3.952	0.083	1.962	3.005	2.727	0.412	1.890	2.425	0.173
Treated by (DFO)	2.334	0.922	3.284	2.202	2.453	3.670	0.066	1.726	1.473	1.232	0.621	0.091	2.331	0.104

Figure S1. Turbiscan Stability Index (TSI) of the tung oil emulsion during the first 8 h. E1, emulsion alone; E2, emulsion diluted in PBS (1:1, v/v); E3, emulsion diluted in PBS (1:1, v/v) + 1% DMSO; E4, emulsion diluted in PBS (1:1, v/v) + AAPH (1 mM); E5, emulsion diluted in PBS (1:1, v/v) + AAPH (1 mM) + Trolox (2 µM).

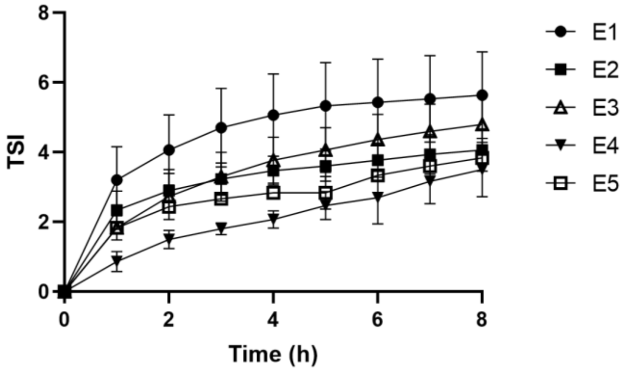

Figure S2. Characterization of the emulsion by dynamic light scattering. A, Z-average main; B, peak size; C, polydispersity index; E1, emulsion alone; E2, emulsion diluted in PBS (1:1, v/v); E3, emulsion diluted in PBS (1:1, v/v) + 1% DMSO; E4, emulsion diluted in PBS (1:1, v/v) + AAPH (1 mM); E5, emulsion diluted in PBS (1:1, v/v) + AAPH (1 mM) + Trolox (2 µM).

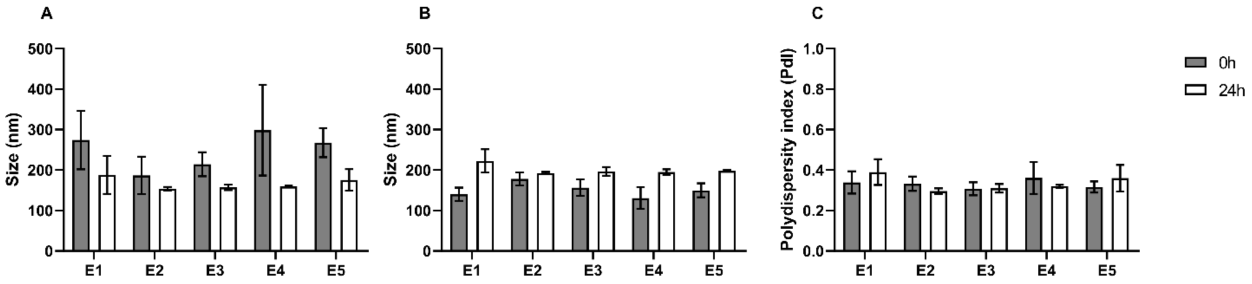

Figure S3. ^{1}H NMR spectra of the studied fractions. The signals corresponding to the main compounds present in each fraction were assigned as follows: (1) δ-amplexichromanol, (2) δ-garcinoic acid, (3) sieboldin, (4) (-)-epicatechin. Spectra A and B were recorded in $CDCl_3$, whereas spectra C and D were recorded in CD_3OD.

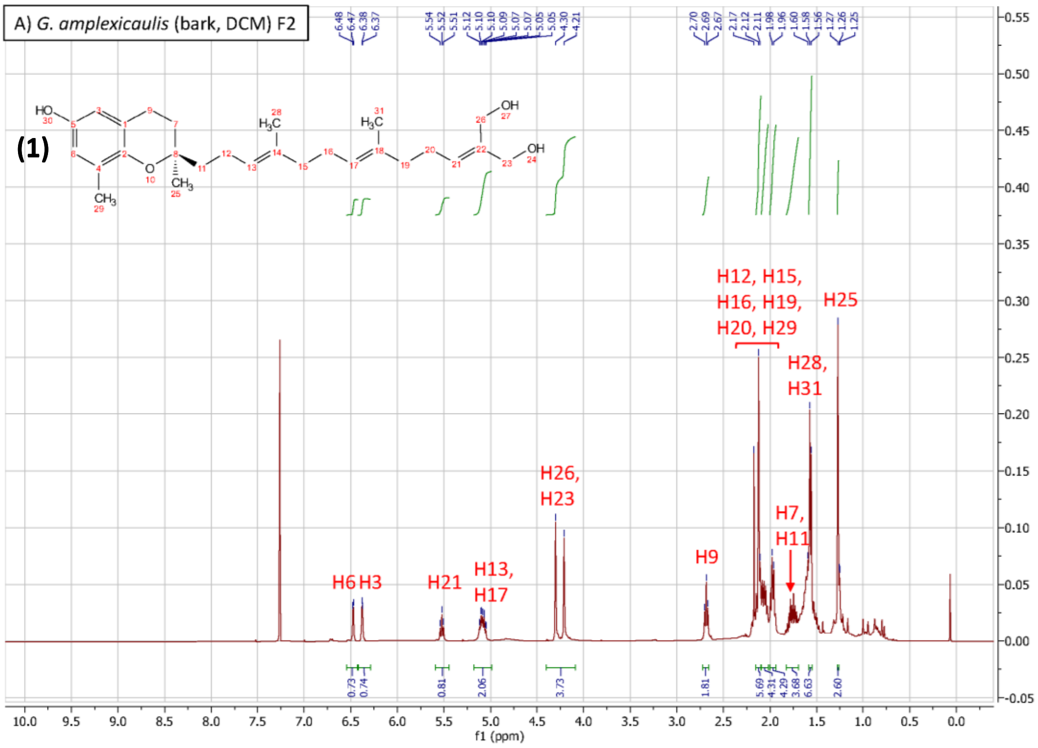

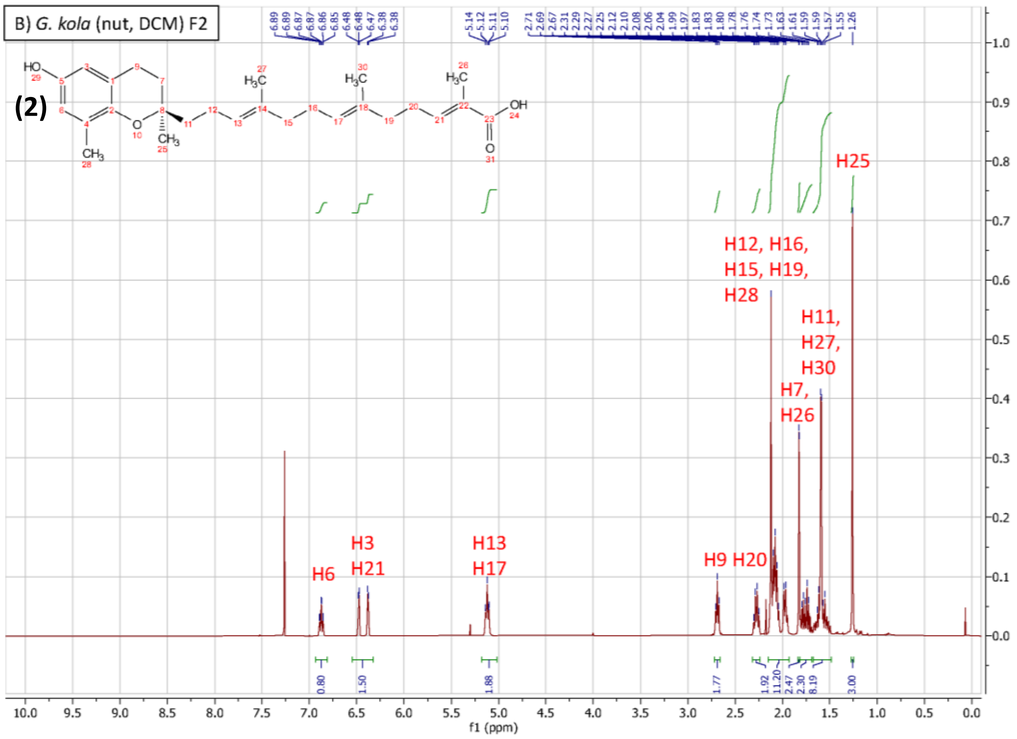

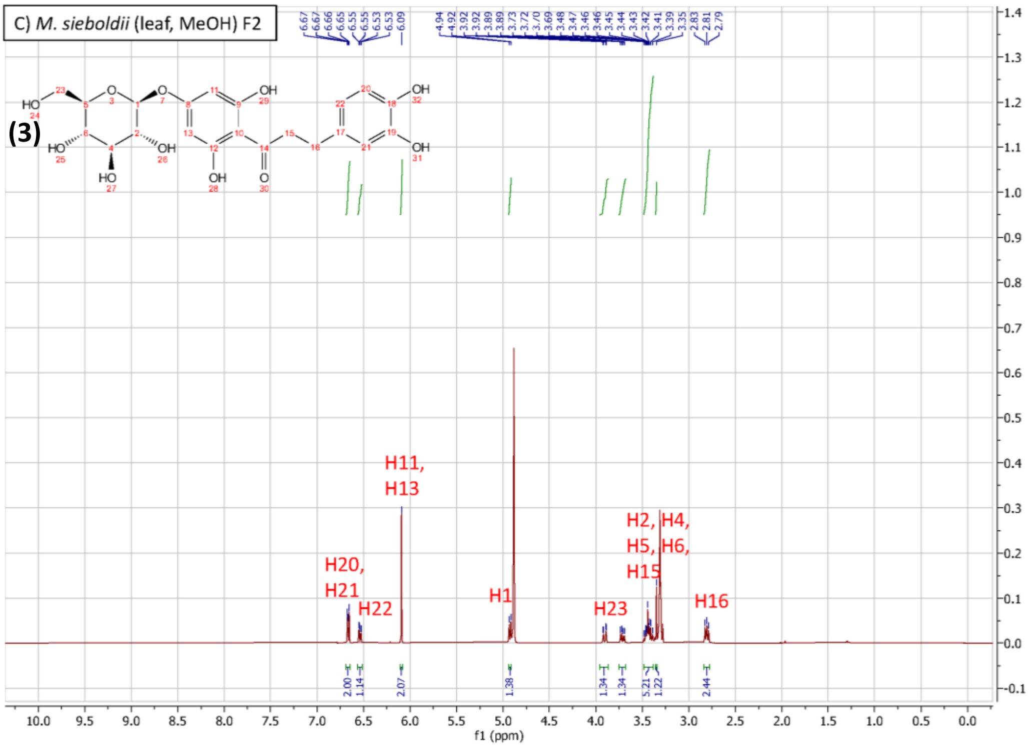

C) *M. sieboldii* (leaf, MeOH) F2
(3)
H11,
H13
H20,
H21
H22
H1
H23
H2, H4,
H5, H6,
H15
H16
f1 (ppm)

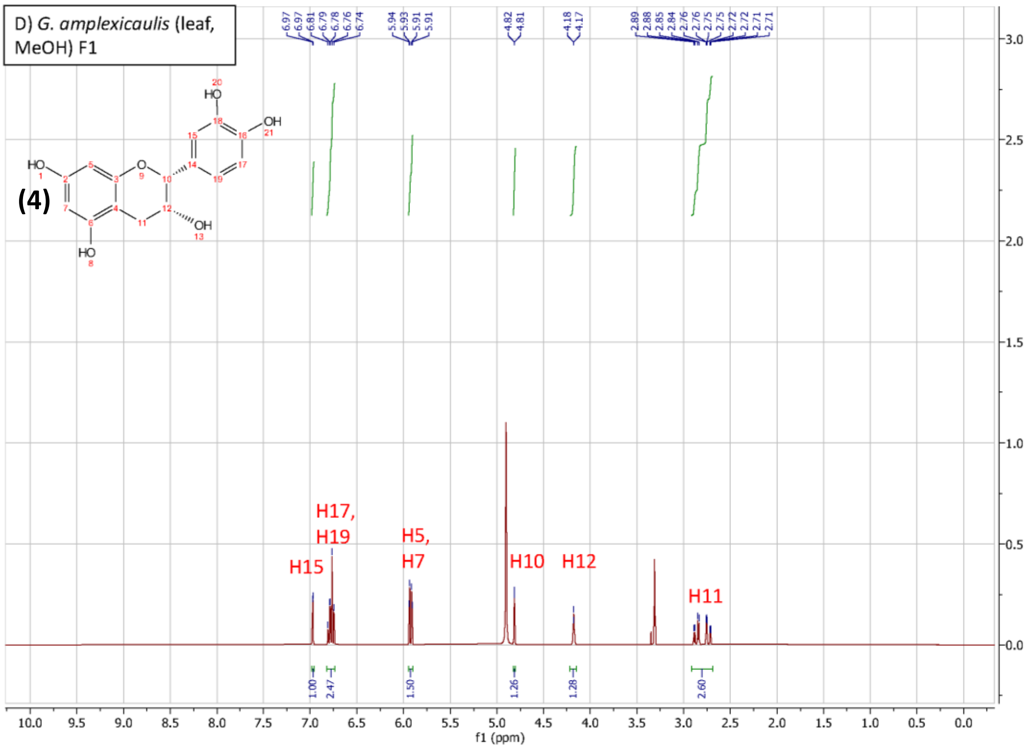

D) *G. amplexicaulis* (leaf, MeOH) F1
(4)
H17,
H19
H15
H5,
H7
H10
H12
H11
f1 (ppm)

Figure S4. HPLC-UV chromatographic profiles of the most active fractions obtained from the four selected extracts. Analyses were performed by HPLC-UV at 280 nm on a Luna C18 column using a water/methanol gradient. For fractions A and B, the mobile phase consisted of water and methanol, with a gradient from 50% to 100% methanol over 15 min, followed by 15 min at 100% methanol and re-equilibration for 5 min at 50% methanol. For fractions C and D, the mobile phase consisted of water and methanol, with a gradient from 5% to 95% methanol over 30 min, followed by 5 min at 100% methanol and re-equilibration for 5 min at 5% methanol. Numbers indicate the compounds previously identified by 1H NMR and assigned to the corresponding HPLC-UV peaks based on comparison with the reference chemical library: (1) δ-amplexichromanol, (2) δ-garcinoic acid, (3) sieboldin, (4) (-)-epicatechin.

A) *G. amplexicaulis* (bark, DCM) fraction 2

(1)

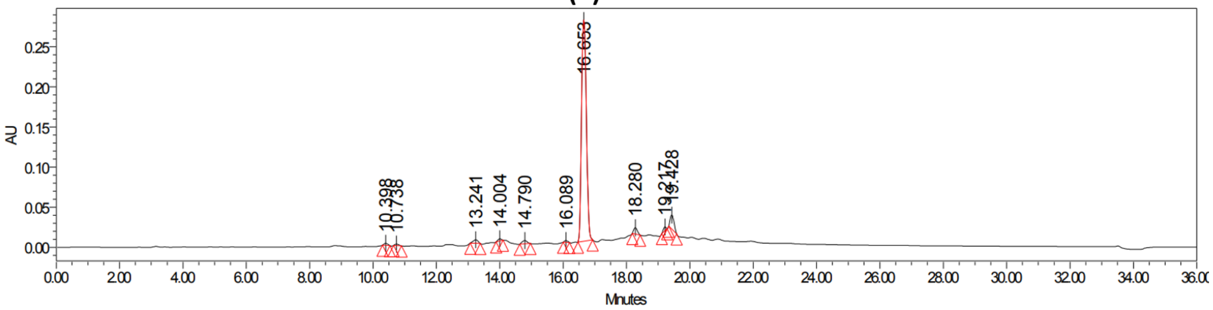

Retention Time	Area	% Area	Height
10.398	21252	0.65	2526
10.738	15833	0.48	1831
13.241	47784	1.45	4428
14.004	19806	0.60	2631
14.790	44198	1.34	4019
16.089	14756	0.45	2002
16.653	2886197	87.66	276302
18.280	63221	1.92	7686
19.217	29554	0.90	4633
19.428	149711	4.55	17644

B) *G. kola* (nut, DCM) fraction 2

(2)

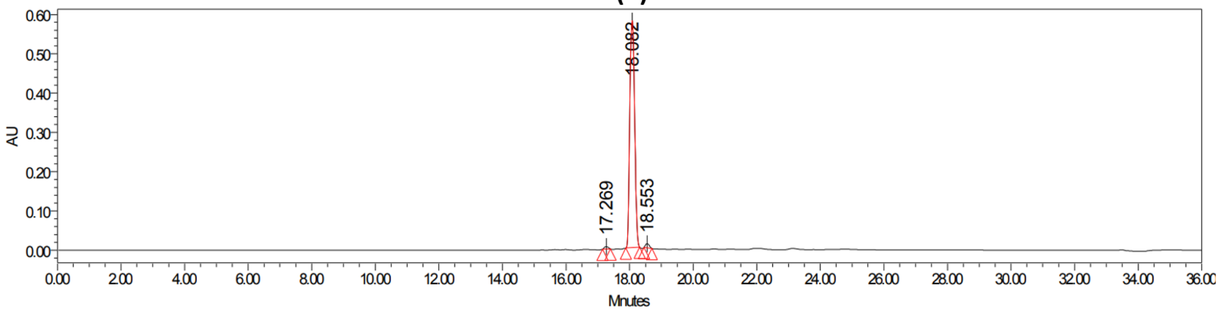

Retention Time	Area	% Area	Height
17.269	56696	0.91	6387
18.082	6092867	97.59	577878
18.553	94001	1.51	10727

C) *M. sieboldii* (leaf, MeOH) fraction 2

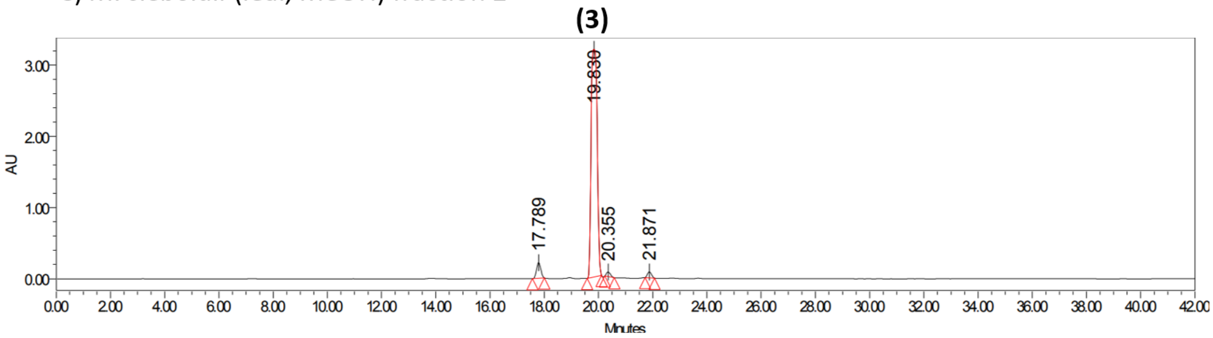

Retention Time	Area	% Area	Height
17.789	2405127	4.42	220148
19.830	50431935	92.77	3198470
20.355	659528	1.21	63787
21.871	864597	1.59	83326

D) *G. amplexicaulis* (leaf, MeOH) fraction 1

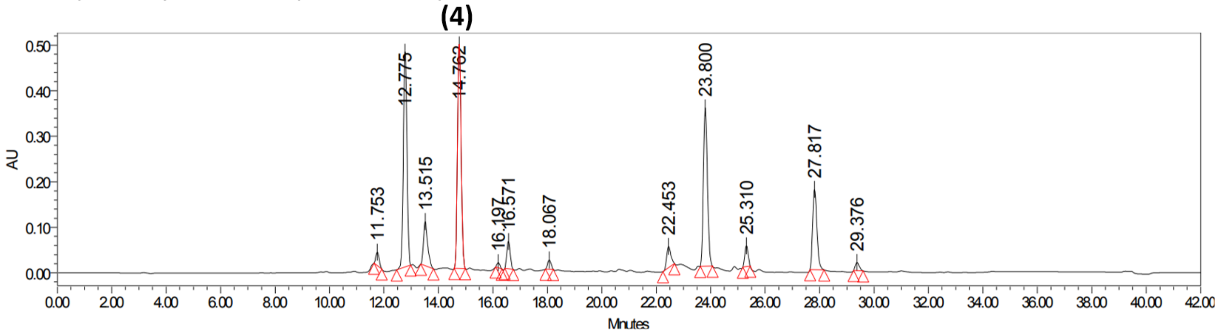

Retention Time	Area	% Area	Height
11.753	256410	1.46	29930
12.775	4398501	24.99	475262
13.515	1042199	5.92	96938
14.762	4530127	25.74	493067
16.197	87979	0.50	11399
16.571	531361	3.02	60240
18.067	180341	1.02	20100
22.453	492445	2.80	47290
23.800	3595676	20.43	347551
25.310	396103	2.25	45926
27.817	1908282	10.84	176322
29.376	178302	1.01	17524

Figure S5. Comparative HPLC-UV chromatographic profiles of G. amplexicaulis (leaf, MeOH) extract, (-)-epicatechin, and δ-amplexichromanol. Analyses were performed by HPLC-UV at 280 nm on a Luna C18 column using a water/methanol gradient. The mobile phase consisted of water and methanol, with elution from 5% to 95% methanol over 30 min, followed by 5 min at 100% methanol and re-equilibration for 5 min at 5% methanol. The chromatographic profile indicates the presence of (-)-epicatechin in the *G. amplexicaulis* (leaf, MeOH) extract, whereas δ-amplexichromanol was not detected under these conditions.

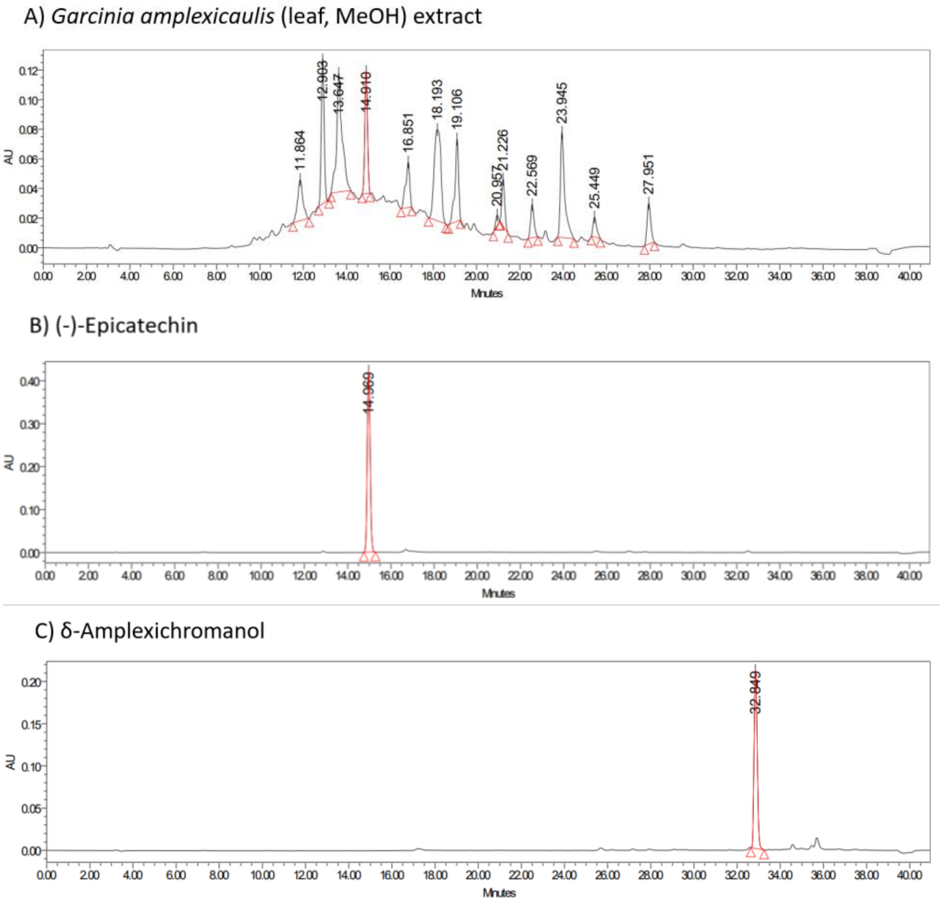

Supplement: Supplementary file 1 [file metabolites-16-00714-s001.zip › metabolites-4470497-supplementary.pdf]
